# Occurrence and Drivers of Antibiotic Resistance Genes Carried by Bacteriophages in Soils Following Different Fertilization Treatments

**DOI:** 10.3390/toxics13060495

**Published:** 2025-06-13

**Authors:** Mingdi Zhang, Yajie Guo, Yue Zhang, Xueying Hu, Shoutao Cheng, Xuming Wang

**Affiliations:** 1 College of Food Science and Engineering, Jilin University, Changchun 130062, China; zhangmd@jlu.edu.cn; 2 Beijing Key Laboratory of Agricultural Genetic Resources and Biotechnology, Institute of Biotechnology, Beijing Academy of Agriculture and Forestry Sciences, Beijing 100097, China; guoyajie@baafs.net.cn (Y.G.); 13001167789@163.com (Y.Z.); 17745161087@163.com (X.H.); chengshoutao@163.com (S.C.); 3 Beijing Da Bei Nong Group, State Key Laboratory of Forage Microbiology Engineering, Beijing 100194, China

**Keywords:** bacteriophages, antibiotic resistance genes, agricultural soil, fertilization, droplet digital PCR

## Abstract

Fertilization has an important effect on soil antibiotic resistance. Most recent studies have focused on antibiotic resistance genes (ARGs) harbored by bacteria (bARGs); however, little is known about ARGs carried by soil bacteriophages (pARGs) under different fertilization treatments. Here, 24 pARG subtypes were quantified in soils with long-term application of different fertilizers using droplet digital PCR (ddPCR). The results showed that the detection rates of the target ARGs in bacteriophages were 66.67%, 70.83%, and 75.00% in unfertilized, chemically fertilized, and organically fertilized soils, respectively. The total abundance of pARGs in soils amended with organic fertilizer was significantly higher than that in unfertilized and chemically fertilized soils. The multidrug resistance gene (*mexF*) exhibited the highest abundance in soils amended with organic fertilizer. A significant positive correlation was observed between bARGs and pARGs, and the detected pARG subtype abundances were one to two orders of magnitude lower than those of the corresponding bARGs. The results of variation partitioning analysis revealed that the interaction between the bacterial community and soil properties drove the variation in soil pARGs. Our findings indicate that bacteriophages are important vectors of ARGs, in addition to bacteria, in agricultural soils, and their contribution to antibiotic resistance should not be overlooked.

## 1. Introduction

Antibiotic resistance constitutes a severe challenge to human health due to the inefficacy of antibiotics in treating infectious diseases in humans and animals [1,2], leading to widespread attention [3,4]. Antibiotic resistance genes (ARGs) have been extensively acknowledged as emerging pollutants and observed in various environmental settings due to the misuse or overuse of antibiotics in clinical settings and animal husbandry. ARGs can be transferred between bacteria via transformation, transduction, and conjugation, leading to the environmental proliferation and dissemination of ARGs. Generally, horizontal gene transfer via plasmid-mediated conjugation represents the primary route for ARG spread, and the contribution of transduction mediated by bacteriophages (phages) to environmental antibiotic resistance remains largely unexplored.

Bacteriophages, ubiquitous in the biosphere, play critical roles in shaping microbial communities, driving biogeochemical processes across ecosystems, and facilitating bacterial evolution [5,6]. Transduction via phages can transfer any DNA fragment, including ARGs, from donor bacteria to recipient bacterial cells. Therefore, phages may play crucial roles in the horizontal transfer of ARGs. However, the relationship between phages and antibiotic resistance has received little attention. Recently, various ARG sequences have been identified in phage genomes from different environmental samples (soil, feces, and water) and foods [7,8,9,10], indicating that phages represent a non-negligible factor in the ecology of antibiotic resistance. Phages can transfer ARGs through transduction. Compared to conjugation, transduction does not require direct cell-to-cell contact between bacteria [11]. Moreover, phages can persist in the environment for extended periods and infect a broad spectrum of bacterial hosts [12,13]. Certain lysogenic phages can integrate ARGs into bacterial chromosomes, facilitating long-term maintenance and vertical transmission of resistance traits [14]. These characteristics suggest that phage-borne ARGs may exhibit a wider host range, higher environmental persistence, and enhanced potential for cross-species transfer compared to those carried by bacterial genomes or plasmids, thereby posing unique ecological and clinical risks.

Soil is one of the largest ARG reservoirs in the environment, and considerable research effort has focused on characterizing the variety and levels of ARGs in soils [15]. However, the distribution and driving mechanisms of ARGs harbored by soil-borne phages have been poorly explored, impeding a better understanding of the mobile antibiotic resistome in the soil. Ross et al. found that all five tested target ARG subtypes (*strA*, *strB*, *sul1*, *aadA*, and *bla_OXA-20_*) were present in phage fractions from soils amended with dairy manure [16]. Anand et al. found occurrence rates of 18.75%, 9.4%, and 3.1% for *bla_TEM_*, *tetW*, and *bla_OXA-2_*, respectively, in 32 phages isolated from soil [7]. Six *tet* genes (*tetC*, *tetE*, *tetG*, *tetM*, *tetO*, and *tetX*) were quantified in phage DNA from greenhouse soils using qPCR technology, and their total abundance reached 8.0 × 10^4^ copies/g [17]. However, these studies have only discussed the diversity and abundance of ARGs harbored by phages, and the mechanisms affecting their occurrence have not been fully explored.

Fertilization, including fertilizer type and dosage, significantly affects the profiles of the soil antibiotic resistome [15,17]; however, its impact on ARGs carried by phages remains largely unknown. Recently, Chen et al. identified 16 unique ARGs carried by soil-borne phages using viral metagenomic sequencing and found that trimethoprim resistance genes were dominant in all samples amended with different fertilizers [18]. Although the existence of ARGs in soil phages has been confirmed, our understanding of their diversity and abundance is incomplete, and absolute quantitative information on the effects of the long-term application of different fertilizers on ARGs in soil phages is lacking. Therefore, we collected soil samples after the long-term application of different fertilizers and investigated the quantitative profiles and driving factors of ARGs harbored by phages using droplet digital PCR (ddPCR). This study provides a scientific foundation for further expanding our knowledge of the soil mobile resistome and controlling the spread of ARGs mediated by phages in agricultural soils.

## 2. Materials and Methods

### 2.1. Experimental Design

The experimental site was set up in a greenhouse (61 m × 8 m) in Daxing District, Beijing (39°26′ N, 116°13′ E), which began in 2009. Eggplants were planted annually from February to July, and sweet peppers were planted from August to January of the following year. The soil was subjected to the following four treatments: without fertilizer (CK), with organic fertilizer (OF), with inorganic fertilizer (IF), and with both organic and inorganic fertilizer (MF). The organic fertilizer is commercially available and derived from chicken manure. The fertilization amounts for each treatment are listed in Table S1. A randomized block design was used for all treatments, with three replicate plots for each treatment. An experimental outline is shown in Figure S1.

### 2.2. Sample Collection

A five-point sampling approach was utilized to collect topsoil (0–20 cm) from all four treatment areas in October 2021. Following passage through a 2 mm mesh sieve, each sample was separated into two representative subsamples. One part was air-dried for physiochemical analysis, and another part was stored at −80 °C for soil bacterial and viral DNA extraction.

### 2.3. Soil Physicochemical Analysis

The soil pH, organic matter (OM), total nitrogen (TN), available phosphorus (AP), available potassium (AK), and heavy metals (As, Hg, Cu, Cr, Pb, Zn, and Cd) were measured according to our previous work [19].

### 2.4. Total DNA Extraction and 16S rRNA Gene Sequencing

Employing the FastDNA Spin kit (MP Biomedical, Rouen, France), total microbial DNA was extracted from 0.5 g of soil following the supplier’s recommended protocol, and the concentration of DNA was measured using a Qubit 3.0 fluorometer (Invitrogen, Carlsbad, CA, USA). The hypervariable regions V3-V4 of the 16S rRNA gene were amplified using PCR by specific primers (338F and 806R) for bacteria as barcodes [20]. The PCR product was purified and sequenced using an Illumina MiSeq PE300 instrument in accordance with Majorbio Bio-Pharm Technology Co. Ltd.’s established protocols (Shanghai, China). The raw reads were deposited in the NCBI Sequence Read Archive database (Accession Number PRJNA1140442). Reads were assigned operational taxonomic units (OTUs) at a 97% similarity cutoff. Community structure was analyzed at the phylum and genus levels using the Silva v138 database with a confidence threshold 0.7 [21].

### 2.5. Bacterial and Phage DNA Extraction and ARG Quantification

The DNA extraction was based on the method described by Chen et al. [18]. Twenty gram soil was homogenized with 25 mL phosphate buffered saline solution (PBS; 0.01 M, pH = 7.0); then, mitomycin C was supplemented to achieve a final concentration of 1 µg/mL [8]. The mixture was suspended by magnetic stirring for 1 h and incubated overnight at 28 °C in the dark for 50 rpm, vortex for 1 h, and then the supernatant was obtained after centrifuging for 10 min at 3000× *g* at 4 °C. Low protein-binding (0.22 µm) was used to retain soil bacteria, and the bacterial DNA was extracted using a FastDNA Spin kit for soil (MP Biomedical, Rouen, France). The filtrate, containing phage particles, was concentrated into 1 mL using 100 kDa Amincon Ultra centrifugal filter units (Millipore, Billerica, MA, USA) and subsequently refiltered by a sterile 0.22 µm Millex-GP filter (Millipore, Billerica, MA, USA). DNase I (Solarbio, Beijing, China) was added into the obtained phage concentrates to make the final concentration of 100 U/mL and incubated at 37 °C for 2 h to degrade the residual DNA derived from host bacteria. DNase I was then inactivated with EDTA (0.5 mol/L, pH 8.0) at 65 °C for 5 min. To ensure that the phage concentrate was free from bacterial DNA contamination, a PCR assay for bacterial 16S rRNA gene was performed for an aliquot of each sample using the universal primers 27F/1492R. Only negative samples (without bacterial DNA) were selected for DNA extraction using the TIANamp Virus DNA/RNA Kit (Tiangen, Beijing, China).

Quantification of ARG abundances carried by bacteria (bARGs) and phages (pARGs) were performed via droplet digital PCR (ddPCR) according to Yin et al. and Morella et al. [22,23]. The ddPCR mixtures included 12.5 mL of EverGreen Supermix (Bio-Rad, Hercules, CA, USA), 0.25 mL of the forward and reverse primers, and 1.25 mL of the DNA template, and sterile water was added to reach a final volume of 25 mL. Sterile water was used as a negative control. The prepared reaction mixture was subjected to droplet generation (12,000–20,000 droplets) using the QX200 Droplet Generator Bio-Rad, Hercules, CA, USA). Subsequently, the generated droplets were transferred to a 96-well plate for PCR amplification on a MyCycler thermal cycler (Bio-Rad, Hercules, CA, USA). Finally, droplet enumeration and data analysis were conducted with the QX200 Droplet Reader and QuantaSoft analysis software (Bio-Rad, Hercules, CA, USA).

Twenty-four ARG subtypes against aminoglycosides (*strA*, *strB*, *aadA-01*), β-lactams (*bla_OXA-20_*, *bla_TEM_*, *bla_CTX-M_*), macrolide–lincosamide–streptogramin B (MLSB) (*ermA*, *ermB*, *mphA-01*, *oleC*), sulfonamides (*sul1*, *sul2*), tetracyclines (*tetA*, *tetW*, *tetM*, *tetX*), quinolones (*qnrA*, *qnrS*), vancomycin (*vanHB*, *vanA*), and multi-drug resistance genes (*acrA-05*, *emrD*, *mepA*, *mexF*) were quantitatively measured using ddPCR technology in the following reaction conditions: 94 °C for 5 min; 39 cycles at 94 °C for 30 s, 60 °C for 30 s, and 72 °C for 1 min; and 72 °C for 10 min, after which the reaction was held at 4 °C. The primers used for ddPCR are shown in Table S2 [24,25,26,27,28].

### 2.6. Statistical Analysis

All data used in the analysis were derived from three biological replicates to ensure robustness and reproducibility of the results. Data averages and relative standard deviations (RSD) were computed with Microsoft Excel 2019 (Microsoft, Redmond, WA, USA). Statistical significance was evaluated using univariate analysis of variance at *p* < 0.05 in IBM SPSS Statistics25 (IBM, Armonk, NY, USA). Bar charts and linear regression analyses were performed using Origin 2018 software (OriginLab Corporation, Northampton, MA, USA). Restricted principal coordinate analysis (PCoA) and redundancy analysis (RDA) plots were conducted online “http://www.ehbio.com/ImageGP/ (accessed on 15 July 2023)”. Variation partitioning analysis (VPA) and heat maps were created online “http://www.cloudtutu.com/ (accessed on 15 July 2023). A co-occurrence network plot was generated using Gephi software (0.9.2). All figures were typographic and formatted using Adobe Illustrator CC2019 (Adobe, San Jose, CA, USA).

## 3. Results and Discussion

### 3.1. Diversity and Abundance of pARGs in Different Fertilization Treatment Soils

A total of 19 pARGs were detected among 24 target genes in the soils with different fertilization treatments, including genes conferring resistance to vancomycin (two subtypes), quinolones (one subtype), multi-drug (four subtypes), tetracyclines (four subtypes), sulfonamides (one subtype), MLSB (two subtypes), β-lactams (two subtypes), and aminoglycosides (three subtypes). The pARGs detected in the CK, IF, OF, and MF treatments were 16, 17, 18, and 18, respectively (Table S3). The highest detection rates were observed for the OF (75.00%) and MF (75.00%) treatments, followed by IF (70.83%) and CK (66.67%). These results indicated that applying organic fertilizers led to an increase in the diversity of soil pARGs. Chen et al. also found that the number of phage-associated ARG subtypes in soil amended with sewage sludge was higher than that in the non-fertilized treatment [18]. Additionally, five ARG subtypes (*bla_OXA-20_*, *ermA*, *ermB*, *sul1*, and *qnrA*) were not detected in soil phages (Table S3).

**Figure 1:**
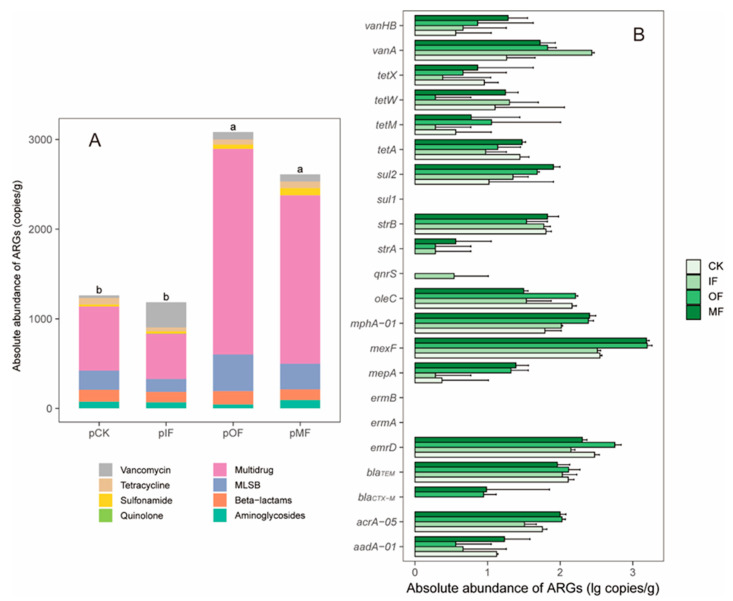
Abundance of pARGs in the soils with four different fertilization treatments. (**A**) Total abundance of pARGs. (**B**) Abundance of different pARG subtypes. CK: without fertilizer; IF: amendment with inorganic fertilizer; OF: amendment with inorganic fertilizer; MF: amendment with mixed organic and inorganic fertilizers.

The total abundance of pARGs in soils amended with organic fertilizer (OF and MF) exhibited significantly greater values compared to the unfertilized and chemically fertilized soils (*p* < 0.05), and multi-drug resistance genes exhibited the highest abundance among all soil samples (Figure 1A). A recent study also showed that the abundance of pARGs increased markedly in soil amended with pig manure compared to that in non-manured soil [29]. As shown in Figure 1B, the dominant pARG subtypes in CK were *mexF*, *emrD*, *oleC* and *bla_TEM_*, with the abundances of 3.53 × 10^2^, 3.00 × 10^2^, 1.47 × 10^2^, and 1.31 × 10^2^ copies/g, respectively. The *mexF* (3.27 × 10^2^ copies/g) and *vanA* (2.73 × 10^2^ copies/g) exhibited higher abundances than other pARGs in IF. Multi-drug resistance genes (*mexF* and *emrD*) were dominant in the soil samples amended with organic fertilizers (OF and MF). The subtype *mexF* exhibited the highest abundance in OF (1.59 × 10^3^ copies/g) and MF (1.55 × 10^3^ copies/g). The abundance of *mphA-01*, *sul2*, *mexF*, and *vanHB* significantly increased in the OF and MF treatments compared to that in the CK (*p* < 0.05). In contrast, some pARGs did not change significantly with fertilization, including *strB*, *bla_TEM_* and *tetX*. Overall, the application of fertilizers, especially organic fertilizers (chicken manure-based), increased the diversity and abundance of pARGs. Future studies will investigate the effects of organic fertilizers from different animal sources on pARGs.

### 3.2. Relationship Between bARGs and pARGs

The diversity and abundance of bARGs in different soil samples are shown in Table S3 and Figure S2. Twenty-two bARG subtypes were detected in the soil bacterial DNA, and two bARG subtypes, *bla_OXA-20_* and *qnrA*, were not detected in any of the soil samples. All soil ARGs detected in the phages were observed in bacteria (Table S3). Compared to CK (87.50%), the detection rates of bARGs in the other three treatments were the same (91.67%). The total abundance of bARGs was one to two orders of magnitude higher than pARGs (Figure S2A and Figure 1A). The abundance of detected bARG subtypes was also higher than that of pARGs (Figure S2B and Figure 1B). Notably, one multi-drug resistance gene (*mexF*) was more abundant than the other ARGs in both soil bacteria and phages, indicating a higher risk of horizontal transfer in the soil [2].

**Figure 2:**
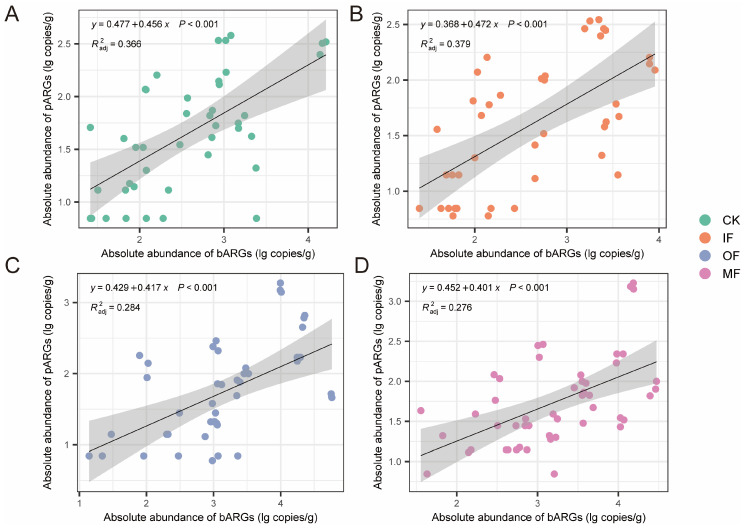
Ordinary least squares (OLS) regression analysis indicating the relationship between bARGs and pARGs in soils with different fertilization treatments: (**A**) CK, (**B**) IF, (**C**) OF, and (**D**) MF.

Ordinary least squares (OLS) regression analysis was performed based on the abundances of pARGs and bARGs (Figure 2). The results showed a significant positive correlation between the abundances of pARGs and bARGs among the four treatments (*p* < 0.001). The highest correlation coefficient between pARGs and bARGs was observed for IF (*R*^2^ = 0.379), followed by CK (*R*^2^ = 0.366), OF (*R*^2^ = 0.284), and MF (*R*^2^ = 0.276). Yang et al. reported a similar positive correlation between pARGs and bARGs in pig farm wastewater treatment plants [30]. This is reasonable because phages can obtain genetic material, including ARGs, from bacteria via transduction [31,32,33].

### 3.3. Co-Occurrence of Bacterial Community and pARGs

**Figure 3:**
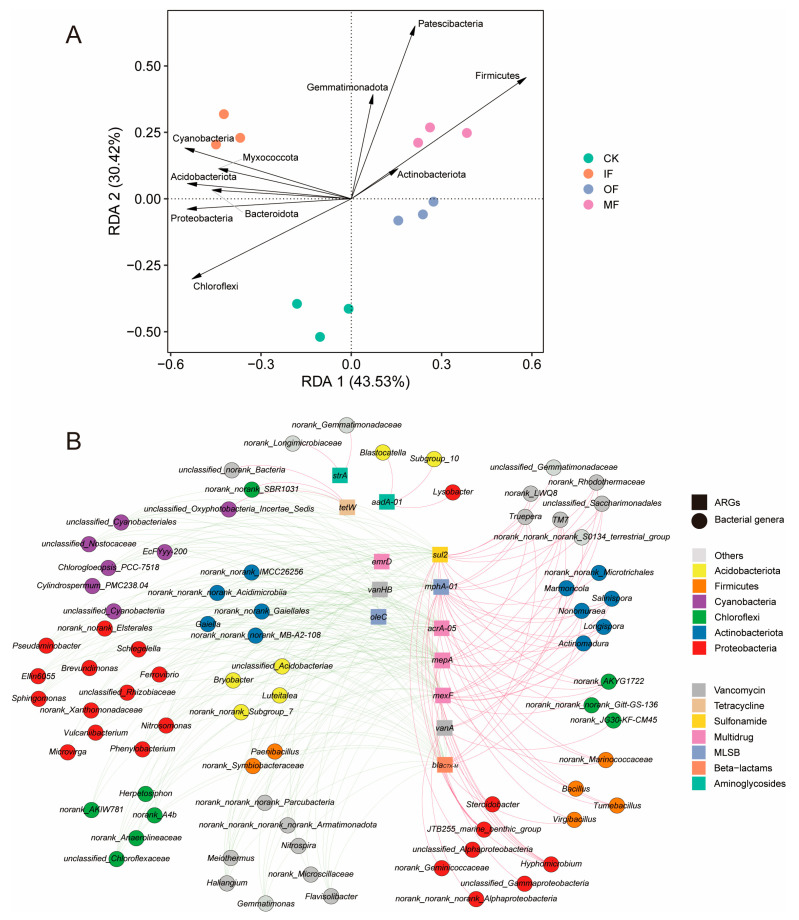
Relationship between pARGs and soil bacterial community. (**A**) Redundancy analysis (RDA) revealing the relationship between pARGs and bacterial community (at the phylum level). (**B**) Co-occurrence pattern of pARGs and bacterial community (at the genus level). Nodes represent pARGs and bacteria genus; edges represent significant correlations (*p* < 0.01, *R* > 0.7 or *R* < −0.7), and edge thickness represent correlation magnitudes.

Many studies have demonstrated that the bacterial community serves as the primary driver affecting ARG profiles [30,34,35], but the relationship between the bacterial community and pARGs remains unclear. The composition of the soil bacterial community is shown in Figure S3. All soil samples were subjected to redundancy analysis (RDA) to identify the relationship between the bacterial community at the phylum level and the pARG profiles (Figure 3A). In the OF and MF treatments, Patescibacteria, Actinobacteria, and Firmicutes were the main drivers of pARGs. Additionally, the co-occurrence patterns between pARGs and bacterial communities at genus level were explored, and the results are shown in Figure 3B. A total of 78 genera exhibited co-occurrence patterns with 13 pARGs, including *aadA-01*, *strA*, *bla_CTX-M_*, *mphA-01*, *oleC*, *acrA-05*, *mepA*, *mexF*, *emrD*, *sul2*, *tetW*, *vanA*, and *vanHB*. Most bacterial genera were significantly and positively correlated with *mphA-01* and *sul2*, followed by *mexF* and *mepA*. The highest number of pARGs (6 subtypes) was significantly associated with *Hyphomicrobium*. Notably, six bacterial genera (*Hyphomicrobium*, *Tumebacbillus*, *Bacillus*, *Streptomyces*, *Microvirga*, and *Gemmatimonas*) exhibited significant co-occurrence patterns with both pARGs and bARGs (Figure S4), indicating that these genera drove the variations of soil total ARGs including bARGs and pARGs. Additionally, these genera were dominant in the soils amended with organic fertilizers (Figure S3), resulting in a high abundance of bARGs and pARGs in the OF and MF treatments (Figure 1 and Figure S2). Previous studies have also reported similar findings, indicating that these genera are known to harbor ARGs and may serve as hosts for bacteriophages, thereby facilitating the spread of ARGs through transduction [36,37,38,39,40,41].

### 3.4. Relationship Between pARGs and Environmental Factors

Applying organic or inorganic fertilizers significantly influenced the heavy metal content in the soil (Table S4). The As, Cu, Cr, and Cd contents in all fertilization treatments were significantly higher than those in CK (*p* < 0.05), but there were no significant differences among OF, IF, and MF (*p* > 0.05). The Zn content in the OF and MF treatments was significantly higher than that in the other two treatments (*p* < 0.05). The application of organic fertilizer aggravated soil acidification [42], and the pH values of OF and MF treatments were lower than those of the other treatments (*p* < 0.05). Compared to IF and CK, the concentrations of OM, AP, AK, and TN were higher in OF and MF (Table S4), which is in accordance with previous studies [43,44].

**Figure 4:**
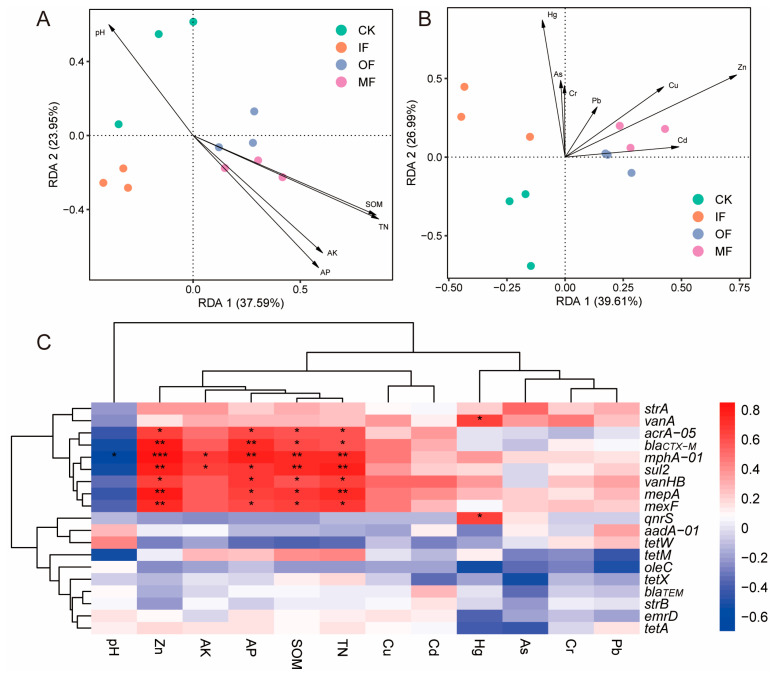
Relationship between pARGs and soil physicochemical properties. (**A**) Redundancy analysis (RDA) reveals the correlation of pARGs with nutritional factors and pH. (**B**) RDA revealing the relationship between pARGs and heavy metals. (**C**) Correlation heatmap of pARGs and physicochemical properties (*, **, *** indicate statistical significance levels below 0.05, 0.01, and 0.001, respectively; red and blue indicating positive and negative correlations, respectively).

RDA was performed to identify the relationship between pARGs and environmental factors. As shown in Figure 4A, the soil pH substantially affected the pARGs in the CK and IF treatments. And the TC, OM, AK, and AP remarkably affected the pARGs in the soils after applying organic fertilizers (OF and MF). The relationship between pARGs and heavy metals showed that Zn, Cu, Pb, Cr, and As influenced pARG profiles (Figure 4B). Cu and Zn primarily drove the distribution of pARGs in soils treated with organic fertilizer (OF and MF), and Cr and As were closely correlated with pARGs in soils amended with inorganic fertilizer (IF). Subsequently, we explored the relationship between the pARG subtypes and environmental factors using Spearman’s coefficient (Figure 4C). The pH exhibited a significant negative correlation with *mphA-01* (*p* < 0.05). The soil nutritional factors, including SOM, TN, AK, and AP, showed significant and positive correlations with various pARGs (*acrA-05*, *bla_CTX-M_*, *mphA-01*, *sul2*, *vanHB*, *mepA*, and *mexF*). This finding is consistent with a previous study [30], which reported that nutritional factors were positively correlated with most pARG subtypes. Soil nutrients can promote microbial activity and growth, potentially leading to higher ARG abundance and increasing rates of horizontal gene transfer, including the transduction of ARGs by bacteriophages [15,16,45]. For heavy metals, Zn exhibited a significantly positive correlation with seven subtypes of pARGs (*acrA-05*, *bla_CTX-M_*, *mphA-01*, *sul2*, *vanHB*, *mepA*, and *mexF*). Early studies have also reported similar results, indicating that heavy metals promote the proliferation of various ARGs [46,47]. The above results show that environmental factors play an important role in pARG variations in fertilized soil.

### 3.5. Contribution of Different Factors to ARG Profile Variation

**Figure 5:**
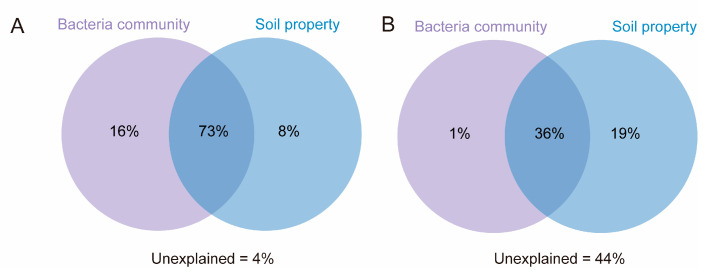
Contribution of influencing factors to bARGs (**A**) and pARGs (**B**).

VPA was performed to explore the contribution of the bacterial community and soil properties (pH, nutrients, and heavy metals) to soil ARG profiles (Figure 5). Soil properties and bacterial communities explained 8% and 16% of the variation in the bARGs, respectively (Figure 5A). The bacterial community and soil properties contributed 1% and 19% of the variation in pARGs, respectively (Figure 5B). Remarkably, interactive contributions were found between the bacterial community and soil properties to bARGs (73%) and pARGs (36%), suggesting that they may be the primary factors determining soil ARGs. However, 44% of pARG variations could not be explained by the selected factors. Previous research has demonstrated that the application of organic fertilizers significantly increased soil antibiotic residues [48], and that pARGs were enriched in high-antibiotic-exposure habitats [14]. Therefore, future studies should explore other factors affecting the distribution of soil pARGs, such as the community structures of soil phages, antibiotic concentrations, soil moisture, and salinity. In addition, we quantitatively determined the abundance of pARGs, and the expression levels of these genes should be investigated through meta transcriptomics technology in future research.

## 4. Conclusions

In this study, we analyzed the abundance and diversity of pARGs in soils subjected to different fertilization treatments. Of the 24 target genes, 19 pARG subtypes were detected, and the application of organic fertilizer (chicken manure-based) increased the diversity and abundance of pARGs. The bacterial community and soil properties drove changes in soil pARGs. Our results highlight the importance of controlling the spread of ARGs carried by phages in agricultural soils.

## Data Availability

The original contributions presented in this study are included in the article/Supplementary Materials. Further inquiries can be directed to the corresponding author.

## Supplementary Materials

The following supporting information can be downloaded at https://www.mdpi.com/article/10.3390/toxics13060495/s1: Figure S1: Experimental idea diagram of the study; Figure S2: The abundance of bARGs in the soils with different fertilization treatments; Figure S3: Characterization of bacterial community in the soils with different fertilization treatments; Figure S4: Network plots between bARGs and bacterial community (genus). Table S1: Fertilizer type and dosage of different treatments; Table S2: PCR primers for ARGs determination using ddPCR; Table S3: Detection of ARGs in the soils with different fertilization treatments; Table S4: Soil physicochemical properties with different fertilization treatments [24,25,26,27,28].
