# Supplementary material for: Occurrence and Drivers of Antibiotic Resistance Genes Carried by Bacteriophages in Soils Following Different Fertilization Treatments
Source: Toxics. 2025 Jun 13;13(6):495. doi: 10.3390/toxics13060495 (PMC12197177; doi:10.3390/toxics13060495)
Supplement: Supplementary file 1 [file toxics-13-00495-s001.zip › toxics-3644364-supplementary.pdf]

Supporting information for

Occurrence and Drivers of Antibiotic Resistance Genes Carried by Bacteriophages in Soils Following Different Fertilization Treatments

Mingdi Zhang [1,†], Yajie Guo [2,†], Yue Zhang [2,3,†], Xueying Hu [2], Shoutao Cheng [2] and Xuming Wang [2,*]

[1] College of Food Science and Engineering, Jilin University, Changchun 130062, China; zhangmd@jlu.edu.cn
[2] Beijing Key Laboratory of Agricultural Genetic Resources and Biotechnology, Institute of Biotechnology, Beijing Academy of Agriculture and Forestry Sciences, Beijing 100097, China; guoyajie@baafs.net.cn (Y.G.); 13001167789@163.com (Y.Z.); 17745161087@163.com (X.H.); chengshoutao@163.com (S.C.)
[3] Beijing Da Bei Nong Group State Key Laboratory of Forage Microbiology Engineering, Beijing 100194, China
* Correspondence: wangxuming@baafs.net.cn; Tel.: +86-10-81127701
† These authors contributed equally to this work.

Tables:

Table S1 Fertilizer type and dosage of different treatments

Table S2 PCR primers for ARGs determination using ddPCR

Table S3 Detection of ARGs in the soils with different fertilization treatments

Table S4 Soil physicochemical properties with different fertilization treatments

Figures:

Fig.S1 Experimental idea diagram of the study

Fig.S2 The abundance of bARGs in the soils with different fertilization treatments. (A)Total abundance of bARGs. (B) The abundance of bARG subtypes.

Fig.S3 Characterization of bacterial community in the soils with different fertilization treatments. (A) ACE index. (B) Shannon index. (C) Relative abundance of dominant bacterial phyla (top 15). (D) Relative abundance of dominant bacterial genera (top 30).

Fig.S4 Network plots between bARGs and bacterial community (genus). Node size represents the number of edges connected to the node, edges represent significant correlations (*P*<0.01, *r*>0.7), and edge thicknesses represent correlation magnitudes.

Table S1. Fertilizer type and dosage of different treatments

Treatment	Fertilizer type and dosage (per year)
Without fertilizer (CK)	--
With inorganic fertilizer (IF)	Urea 0.04 kg/ m^2, $(NH_4)_2HPO_4$ 0.05 kg/ m^2, K_2SO_4 0.03 kg/ m^2
With organic fertilizer (OF)	Compost from chicken manure, 2.38 kg/ m^2
With mixed inorganic and organic fertilizer (MF)	Urea 0.02 kg/ m^2, $(NH_4)_2HPO_4$ 0.025 kg/ m^2, K_2SO_4 0.015 kg/ m^2, compost from chicken manure, 1.20 kg/m^2

Table S2 PCR primers for ARGs determination using ddPCR

ARGs		Primes	Annealing temperature (℃)	References
Aminoglycosides	*strA*	CCGGTGGCATTTGAGAAAAA<br>GTGGCTCAACCTGCGAAAAG	60	(Ouyang et al.,2015)
	strB	GCTCGGTCGTGAGAACAATCT<br>CAATTTCGGTCGCCTGGTAGT	60	(Ouyang et al.,2015)
	aadA-01	GTTGTGCACGACGACATCATT<br>GGCTCGAAGATACCTGCAAGAA	60	(Ouyang et al.,2015)
β-lactams	*bla*$_{OXA-20}$	TGATGATTGTCGAAGCCAAA<br>GCCTGTAGGCCACTCTACCC	60	(Bert,2002)
	bla$_{TEM}$	AGCATCTTACGGATGGCATGA<br>TCCTCCGATCGTTGTCAGAAGT	60	(Ouyang et al.,2015)
	bla$_{CTX-M}$	CAGATTCGGTTCGCTTTCAC<br>GCAAATACTTTATCGTGCTGATG	55	(Yang et al.,2018)
MLSB	*ermA*	TTGAGAAGGGATTTGCGAAAAG<br>ATATCCATCTCCACCATTAATAGTAAACC	60	(Ouyang et al.,2015)
	ermB	TAAAGGGCATTTAACGACGAAACT<br>TTTATACCTCTGTTTGTTAGGGAATTGAA	60	(Ouyang et al.,2015)
	mphA-01	CTGACGCGCTCCGTGTT<br>GGTGGTGCATGGCGATCT	60	(Ouyang et al.,2015)
	oleC	CCCGGAGTCGATGTTCGA<br>GCCGAAGACGTACACGAACAG	60	(Ouyang et al.,2015)
Sulfonamides	*sul1*	CAGCGCTATGCGCTCAAG<br>ATCCCGCTGCGCTGAGT	60	(Ouyang et al.,2015)
	sul2	TCATCTGCCAAACTCGTCGTTA<br>GTCAAAGAACGCCGCAATGT	60	(Ouyang et al.,2015)
Tetracyclines	*tetA*	CTCACCAGCCTGACCTCGAT<br>CACGTTGTTATAGAAGCCGCATAG	60	(Chen et al.,2019)
	tetW	ATGAACATTCCCACCGTTATCTTT<br>ATATCGGCGGAGAGCTTATCC	60	(Chen et al.,2019)
	tetM	GGAGCGATTACAGAATTAGGAAGC<br>TCCATATGTCCTGGCGTGTC	60	(Chen et al.,2019)
	tetX	AAATTTGTTACCGACACGGAAGTT<br>CATAGCTGAAAAAATCCAGGACAGTT	60	(Ouyang et al.,2015)
Multi-drug resistance	*acrA-05*	CGTGCGCGAACGAACA<br>ACTTTGCGCGCCATCTTC	60	(Ouyang et al.,2015)
	emrD	CTCAGCAGTATGGTGGTAAGCATT<br>ACCAGGCGCCGAAGAAC	60	(Ouyang et al.,2015)

	mepA	ATCGGTCGCTCTTCGTTCAC<br>ATAAATAGGATCGAGCTGCTGGAT	60	(Ouyang et al.,2015)
	mexF	CCGCGAGAAGGCCAAGA<br>TTGAGTTCGGCGGTGATGA	60	(Ouyang et al.,2015)
Quinolones	*qnrA*	AGGATTTCTCACGCCAGGATT<br>CCGCTTTCAATGAAACTGCAA	60	(Ouyang et al.,2015)
	qnrS	CGACGTGCTAACTTGCGTGA<br>GGCATTGTTGGAAACTTGCA	60	(Colomer-Lluch et al.,2014)
Vancomycins	*vanHB*	GAGGTTTCCGAGGCGACAA<br>CTCTCGGCGGCAGTCGTAT	60	(Ouyang et al.,2015)
	vanA	GGGCTGTGAGGTCGGTTG<br>TTCAGTACAATGCGGCCGTTA	60	(Chen et al.,2019)

Table S3 Detection of ARGs in the soils with different fertilization treatments

ARG subtypes	Bacteria				Bacteriophages			
	CK	IF	OF	MF	CK	IF	OF	MF
strA	+	+	+	+	—	+	+	+
strB	+	+	+	+	+	+	+	+
aadA-01	+	+	+	+	+	+	+	+
*bla*OXA-20	—	—	—	—	—	—	—	—
*bla*TEM	+	+	+	+	+	+	+	+
*bla*CTX-M	—	+	+	+	—	—	+	+
ermA	+	+	+	+	—	—	—	—
ermB	+	+	+	+	—	—	—	—
mphA-01	+	+	+	+	+	+	+	+
oleC	+	+	+	+	+	+	+	+
sul1	+	+	+	+	—	—	—	—
sul2	+	+	+	+	+	+	+	+
tetA	+	+	+	+	+	+	+	+
tetW	+	+	+	+	+	+	+	+
tetM	+	+	+	+	+	+	+	+
tetX	+	+	+	+	+	+	+	+
acrA-05	+	+	+	+	+	+	+	+
emrD	+	+	+	+	+	+	+	+
mepA	+	+	+	+	+	+	+	+
mexF	+	+	+	+	+	+	+	+
qnrA	—	—	—	—	—	—	—	—
qnrS	+	+	+	+	—	—	—	—
vanHB	+	+	+	+	+	+	+	+
vanA	+	+	+	+	+	+	+	+
Detection rate (%)	87.50	91.67	91.67	91.67	66.67	70.83	75.00	75.00

Note: “+”, detected; “-”, undetected

Table S4 Soil physicochemical properties with different fertilization treatments

Parameters	CK	IF	OF	MF
As (mg/kg)	9.44±0.53b	11.08±0.76a	10.48±0.52a	10.20±1.08ab
Hg (mg/kg)	0.07±0.02a	0.10±0.03a	0.08±0.03a	0.08±0.01a
Cu (mg/kg)	25.92±1.49b	30.65±2.68a	33.06±1.76a	33.23±3.72a
Cr (mg/kg)	66.23±2.36b	75.09±3.65a	72.11±1.23a	72.90±5.36a
Cd (mg/kg)	0.42±0.09b	0.47±0.12a	0.56±0.11a	0.55±0.09a
Pb (mg/kg)	20.66±0.93b	22.49±1.96a	21.84±0.29ab	22.92±0.79a
Zn (mg/kg)	64.60±2.71c	77.10b±6.18c	90.49±10.24ab	104.48±25.29a
pH	8.61±0.19a	8.03±0.17b	7.77±0.14c	7.66±0.22c
OM (g/kg)	10.07±1.15b	13.77±0.98b	31.67±3.83a	34.58±9.53a
AP (mg/kg)	15.33±5.54c	65.45±7.41b	155.90±61.57a	162.30±47.95a
AK (g/kg)	0.12±0.03c	0.32±0.17bc	0.57±0.23b	0.96±0.49a
TN (%)	0.08±0.01b	0.11±0.01b	0.25±0.02a	0.29±0.07a

Note: Different letters in the same row indicating the significant difference ($P<0.05$)

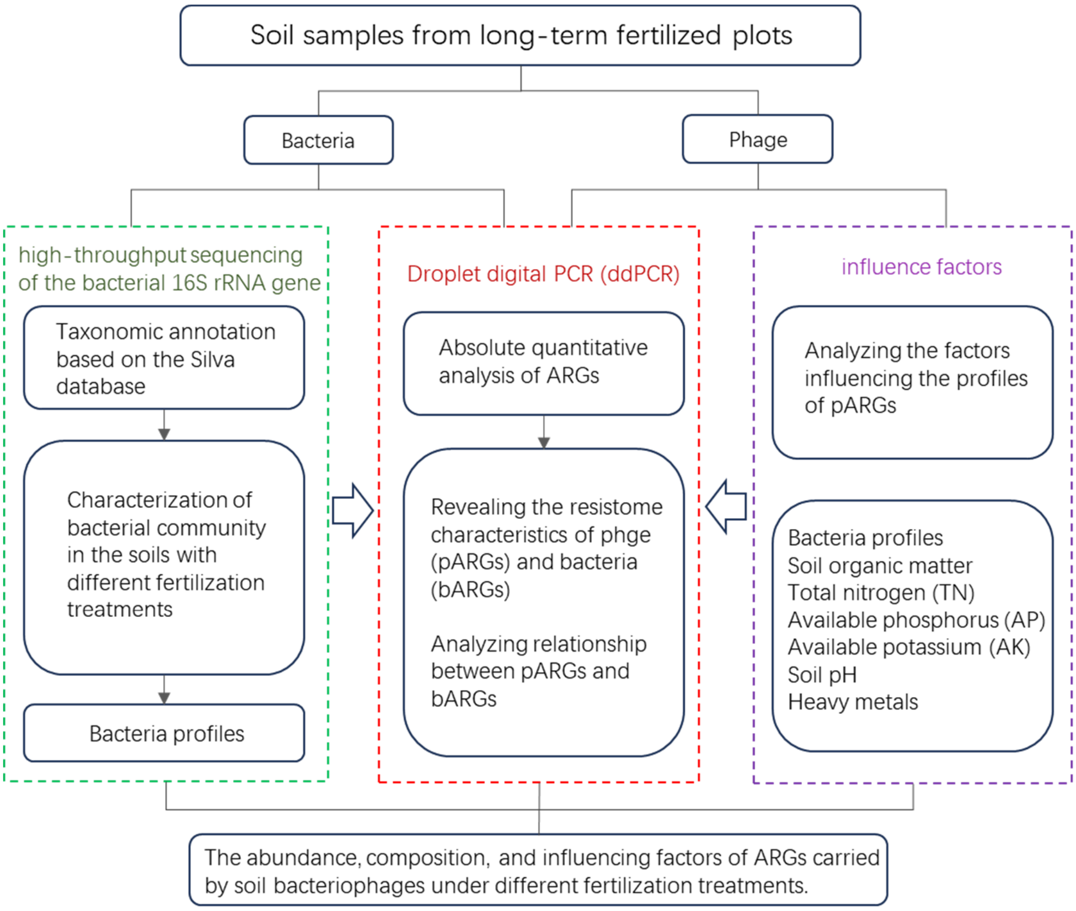

Fig.S1 Experimental idea diagram of the study

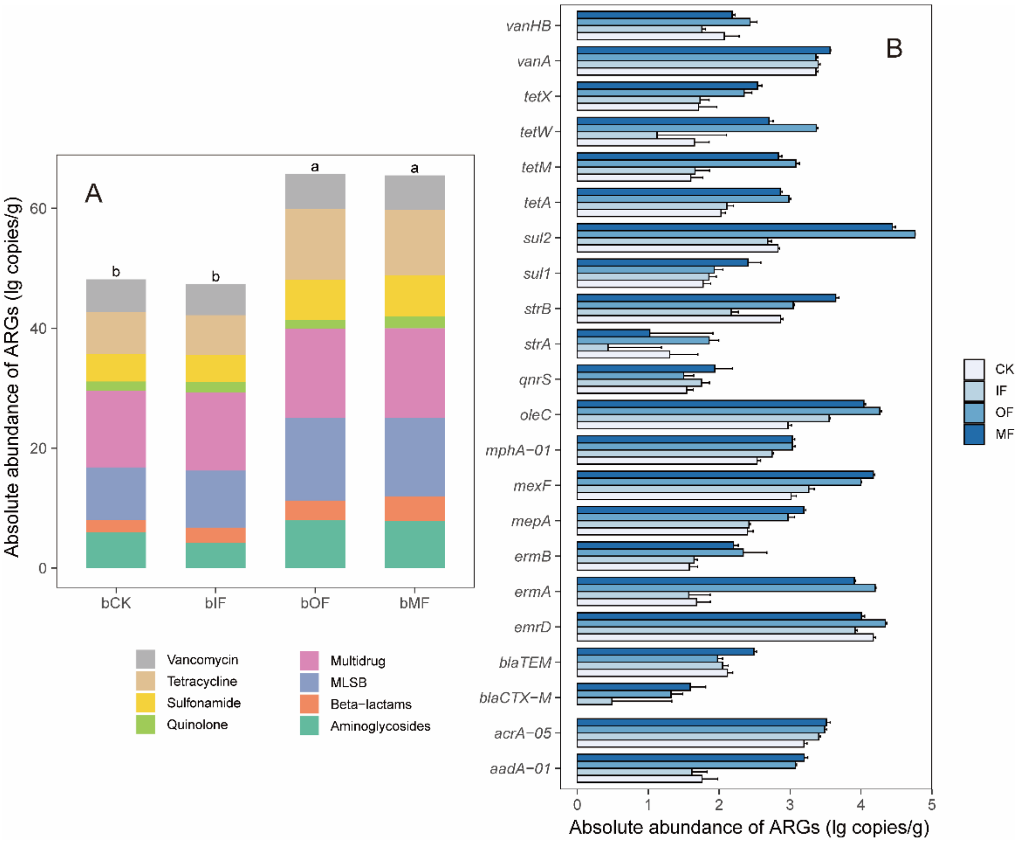

Fig.S2 The abundance of bARGs in the soils with different fertilization treatments. (A)Total abundance of bARGs . (B) The abundance of bARG subtypes.

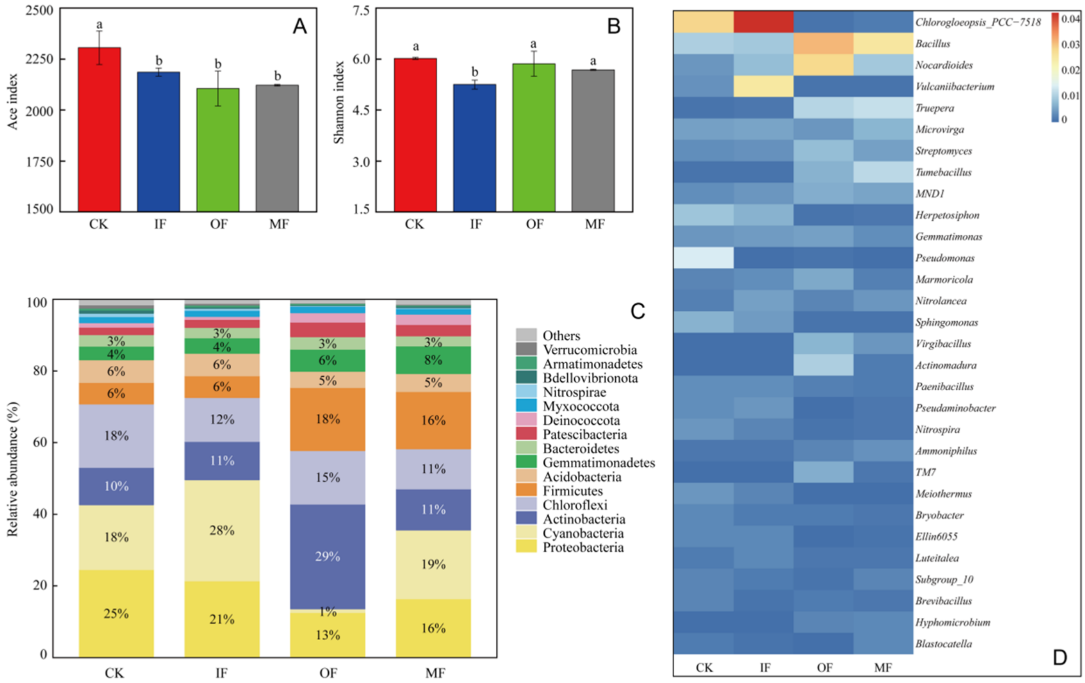

Fig.S3 Characterization of bacterial community in the soils with different fertilization treatments. (A) ACE index. (B) Shannon index. (C) Relative abundance of dominant bacterial phyla (top 15). (D) Relative abundance of dominant bacterial genera (top 30).

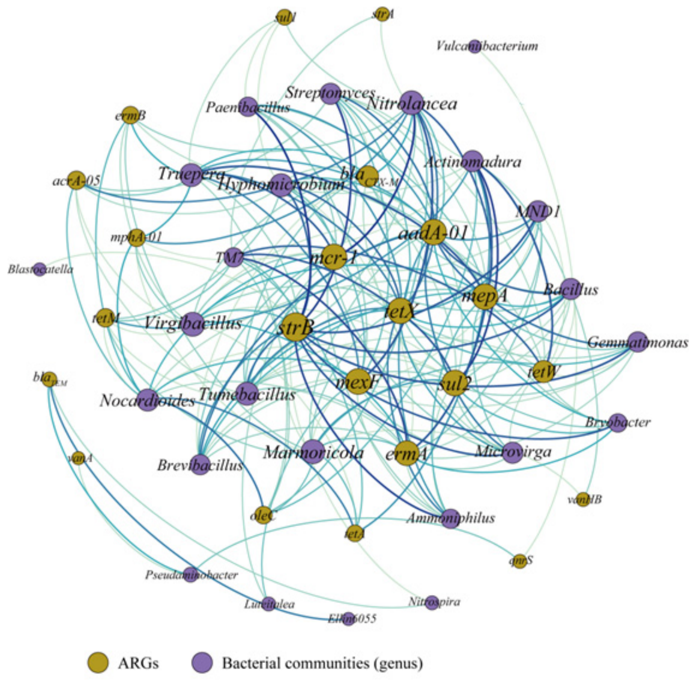

Fig.S4 Network plots between bARGs and bacterial community (genus). Node size represents the number of edges connected to the node, edges represent significant correlations (P<0.01, r>0.7), and edge thicknesses represent correlation magnitudes.

References:

1. Bert, F. Identification of PSE and OXA beta-lactamase genes in *Pseudomonas aeruginosa* using PCR-restriction fragment length polymorphism. *J. Antimicrob. Chemoth.* **2002**, *50*, 11–18. https://doi.org/10.1093/jac/dkf069.
2. Chen, Z.; Zhang, W.; Yang, L.; Stedtfeld, R.D.; Peng, A.; Gu, C.; Boyd, S.A.; Li, H. Antibiotic resistance genes and bacterial communities in cornfield and pasture soils receiving swine and dairy manures. *Environ. Pollut.* **2019**, *248*, 947–957. https://doi.org/10.1016/j.envpol.2019.02.093.
3. Colomer-Lluch, M.; Jofre, J.; Muniesa, M. Quinolone resistance genes (qnrA and qnrS) in bacteriophage particles from wastewater samples and the effect of inducing agents on packaged antibiotic resistance genes. *J. Antimicrob. Chemoth.* **2014**, *69*, 1265–1274. https://doi.org/10.1093/jac/dkt528.
4. Ouyang, W.; Huang, F.; Zhao, Y.; Li, H.; Su, J. Increased levels of antibiotic resistance in urban stream of Jiulongjiang River, China. *Appl. Microbiol. Biot.* **2015**, *99*, 5697–5707. https://doi.org/10.1007/s00253-015-6416-5.
5. Yang, Y.; Shi, W.; Lu, S.; Liu, J.; Liang, H.; Yang, Y.; Duan, G.; Li, Y.; Wang, H.; Zhang, A. Prevalence of antibiotic resistance genes in bacteriophage DNA fraction from Funan River water in Sichuan, China. *Sci. Total Environ.* **2018**, *626*, 835–841. https://doi.org/10.1016/j.scitotenv.2018.01.148.
